# Preparation and physicochemical studies on polymeric nanocomposites containing copper oxide nanoparticles

**DOI:** 10.1080/15685551.2023.2190222

**Published:** 2023-03-15

**Authors:** Soad Alsheheri, Zahra Alamshany, Magdy Y. Abdelaal

**Affiliations:** a Chemistry Department, Faculty of Science, King Abdulaziz University, Jeddah, Saudi Arabia; b Chemistry Department, Faculty of Science, Mansoura University, Mansoura, Egypt

**Keywords:** CMC, PVP, Nano-composite, Copper oxide nanoparticles

## Abstract

The current work aims to modify carboxymethyl cellulose (CMC) and polyvinylpyrrolidone (PVP) with copper oxide nanoparticles (CuO NPs) to obtain new nanocomposites of CMC, PVP, and CuO NPs (CMC/PVP/CuO NPs) with distinguished properties. The interaction between the components of the nanocomposites was suggested and supported by using Gaussian 09W 07 Software and the average particle size was manually determined from TEM images using ImageJ software developed at the National Institutes of Health (NIH). The preparation methods were optimized, and the obtained nanocomposites were characterized with suitable techniques to explore their characteristics and to help expect or predict the suitable fields of applications.

## 1. Introduction

Polymeric nanocomposites can be considered as materials with different amounts of nanoparticles as fillers in the target polymers [1,2]. As the distribution homogeneity of such nano-materials is high within the nano-composite, the interaction of the nano-materials with the polymer matrix would be high too [3,4]. In addition, nanocomposites show remarkable characteristics in many directions, such as electrical, optical, biocompatibility, and biodegradability, which are beneficial in industrial, medical, drug release, packaging, and agricultural applications [5,6]. Carboxymethyl cellulose (CMC) showed high viscosity in water and great compatibility with other water-soluble materials like glues, softeners, and resin, therefore, it attracts much attention in numerous industrial applications such as paper, pharmaceuticals, food, agriculture, barriers, and other [7–9]. Polyvinyl pyrrolidone (PVP) is a semi-crystalline vinyl polymer with a high glass transition temperature (T*g*) due to the considerable closeness in pyrrolidone groups along the polymeric chains [10,11].

Copper oxides are known to be useful in several ways. They are a p-type semiconductor material with limited band gap due to their ease of preparation, nontoxic nature, and reasonably great electrical and optical properties [12–17]. Copper oxide nanoparticles are of extraordinary interest due to their potential applications in a wide variety of areas including electronic and optoelectronic gadgets, such as field impact transistors, electrochemical cells, gas sensors, and nano-devices for catalysis. Copper oxide nanoparticles are doped in different polymeric materials and investigated for applications such as gas-detecting materials, solar cells, and optical switches [18,19]. Thus, the present work aims to prepare nanocomposites of copper oxide nanoparticles with CMC and PVP polymers followed by characterization and investigation of the impact of copper oxide nanoparticles on the physical and chemical properties of the obtained nanocomposites.

## 2. Experimental part

### 2.1. Materials and techniques

Carboxymethylcellulose (CMC) (MW 41 kDa) was purchased from Alpha Chemika, India. Polyvinyl pyrrolidone (PVP) and other conventional chemicals were purchased from Sigma-Aldrich Co. and used without further purification. Laser ablation to prepare CuO NPs was performed by using PRII 8000 Continuum laser, Electro-optics, Inc. FTIR spectra were recorded on a ThermoScientific Nicolet Spectrometer iS10 FT-IR Spectrometer. UV/Vis Spectroscopy was recorded with the JASCO 630 UV/Vis Spectrometer, Japan. These spectra cover the range from 200 to 800 nm. X-Ray powder diffraction spectroscopy (XRPD) Philips PW150 was employed to record the powder diffraction patterns in the presence of Ni clarified using Cu Kα radiation (λ = 1.540 A°) at 40 kV, 30 mA, and a scanning range of 2θ = 18–80. Scanning electron microscopy (SEM) was used to study the surface and morphology of the nanomaterial. For this purpose, JEOL JSM-6510 LV SEM was used after coating the nanoparticles with a gold thin film before the SEM processing. Transmission electron microscopy (TEM) was employed to observe images and particle sizes of the obtained samples by using a JEOL JEM-2100 transmission electron microscope operating at 120 kV. The samples were prepared by depositing a suspension of fine sample powder onto a copper grid coated with a holey carbon foil and dried at ambient temperature. The average particle size of the generated CuO-NPs was determined manually by analyzing the TEM micrograph using Image Software developed at the National Institutes of Health (NIH) [20].

### 2.2. Preparation of copper oxide nanoparticles (CuO NPs) by laser ablation technique

Copper oxide nanoparticles (CuO NPs) were synthesized successfully by laser ablation technique in aqueous media [21,22]. A high purity copper target of dimension 4 × 4 × 2 (mm) was polished and sonicated before subjecting to a Nd:YAG nanosecond pulsed laser adopting a 1064 nm beam of width 6 nm and power 4 Watt with pulse frequency 10 Hz. Under the experimental conditions of laser ablation, Cu was allowed to be oxidized into CuO, and the copper target was immersed in a double-distilled water vessel, and the obtained CuO NPs were characterized.

### 2.3. Preparation of CMC/PVP/CuO NPs nanocomposites

A solution of CMC was prepared by dissolving 3 g CMC in 300 ml of distilled water, and a solution of PVP was prepared by dissolving 3 g PVP in 60 ml of distilled water. A polymer blend solution of CMC and PVP was prepared by mixing the two previously prepared solutions of polymers at a 1:1 ratio (CMC: PVP) while continuously stirring until a homogeneous viscous liquid was formed. Samples of the nanocomposite were prepared by adding different amounts of CuO NPs to the polymer mixture as listed in Table (1), cast on plastic Petri dishes, and dried in an oven at 50 °C for 24 h.

**Table 1.** Composition of CMC/PVP/CuO NPs nanocomposite samples.

Sample	CMC Wt%	PVP Wt%	CuO ml	NC1	NC2	NC3	NC4	NC5	NC6
CMC	100	-	-	50	50	50	50	50	50
PVP	-	100	-	50	50	50	50	50	50
CuO	-	-	100	-	2	4	8	10	12

### 2.4. Characterization of the obtained CMC/PVP/CuO NPs nanocomposites

The prepared samples of polymer nanocomposites were characterized by using XRD, FT-TR, and UV/Vis. Spectroscopy as well as Scanning and Transmittance Electron Microscope (SEM & TEM).

### 2.5. The anti-bacterial activity measurements

The antibacterial activity of the investigated nanocomposites was tested against Gram Positive bacteria (*Staphylococcus aureus*) and Gram-Negative bacteria (*Escherichia coli*). Each of the nanocomposites was dissolved in DMSO, and a solution of 1 mg/mL concentration was prepared separately. Paper discs of Whatman filter paper were prepared with standard size (5 cm) were cut and sterilized in an autoclave. The paper discs soaked in the tested solutions were placed aseptically in Petri dishes containing nutrient agar media (Agar 20 g + beef extract 3 g + peptone 5 g) seeded with *Staphylococcus aureus* and *E. coli*. Petri dishes were incubated at 36 °C and inhibition zones were recorded after 24 h of incubation. Each treatment was replicated three times. The antibacterial activity of a common standard antibiotic Amoxycillin was also recorded under the same conditions. The % activity index was calculated by Eq. 1 [23,24].

(1) $$\%\;Activity\;Index=\;\frac{Inhibition\;Zone\;Diameter\;of\;Sample}{Inhibition\;Zone\;Diameter\;of\;Standard}\times 100$$

## 3. Results and discussion

### 3.1. X-Ray spectroscopy

**Figure 1.**
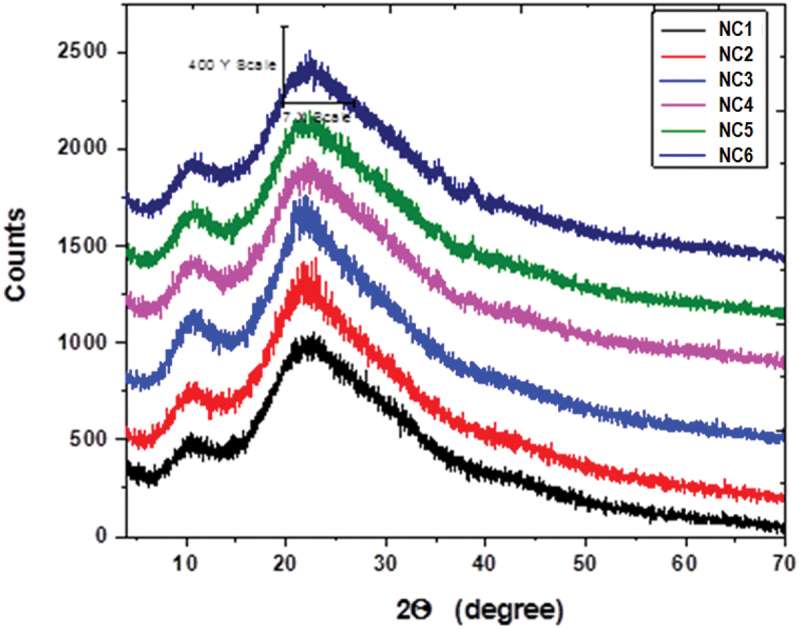
XRPD patterns of CMC/PVP blend **(NC1)** and its CuO NPs nanocomposites **(NC2-NC6)**.

X-Ray powder diffraction (XRPD) patterns were recorded. Figure (1) shows XRPD pattern of the studied samples (**NC1-NC6**). **NC1** shows one broad diffraction peak centered at 2θ = 22.60° related to the amorphous phase of CMC beside 2 main peaks at 2θ = 11° and 21° related to the amorphous PVP [25–29]. The XRD patterns of the investigated CMC/PVP/CuO NPs nanocomposites (**NC2-NC6**) show nearly parallel curves with a small deviation in both intensity (I) and full width at half maximum (FWHM) as summarized in Table 2. This indicates that the change in crystallinity of the nanocomposites is correlated with the amount of CuO-NPs included. The obtained data point to an increase in the amorphous nature of the nanocomposites with increasing CuO-NPs content. This may be attributed to the interstitial position of CuO-NPs within the polymeric matrix at random and/or specific positions that result from the distribution of the nanoparticles within the polymeric matrix. This will be reflected, of course, on most of the physical characteristics of the nanocomposites.

**Table 2.** The intensity (I) and full width at half maximum (FWHM) of the samples.

Sample	NC1	NC2	NC3	NC4	NC5	NC6
I	652	868	887	731	792	872
FWHM	18	20	20	19	22	24

### 3.2. Transmission electron microscopy (TEM)

**Figure 2.**
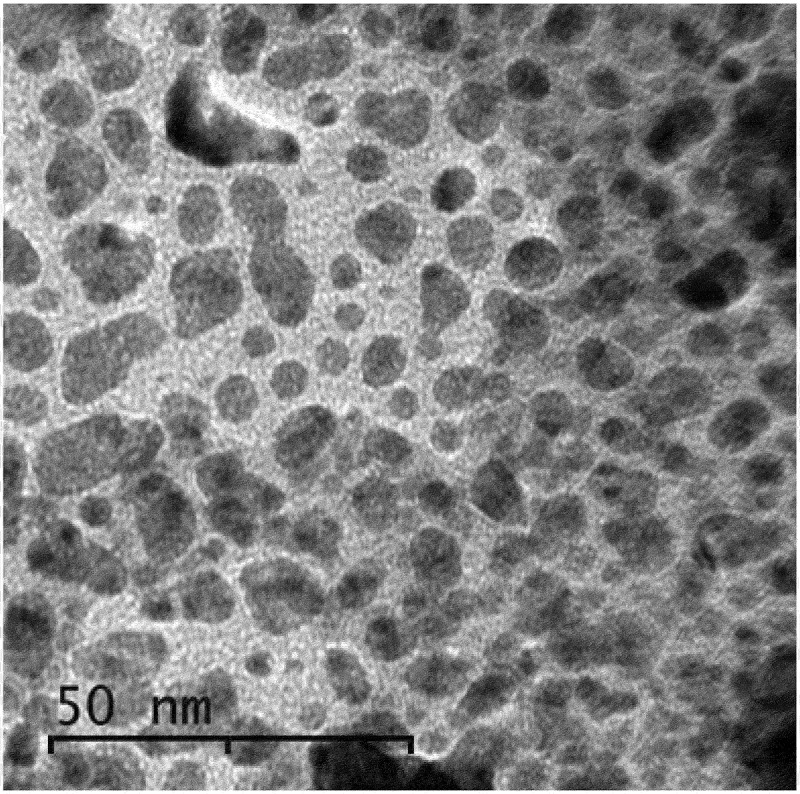
TEM image of CuO NPs prepared by laser ablation.

Images and particle sizes were observed by TEM using a JEOL JEM-2100 Transmission Electron Microscope operating at 120 kV. The samples were prepared by depositing a suspension of the fine sample powder onto a copper grid coated with a holey carbon foil and dried at ambient temperature. TEM image of the prepared CuO NPs shown in Figure (2) indicated more or less distorted spherical shape domains with a slight deviation in shape and particle size. The average particle size of the generated CuO NPs was calculated by averaging approx. 121 particles from TEM images using ImageJ software developed at the National Institutes of Health [30]. The data derived from the image analysis is summarized in Table 3. The average particle diameter **(D)** can be calculated from the average particle size after the approximation that the particles are quasi-spherical in shape according to Eq. 2.

(2) $$\begin{array}{rl} & Sphere\;Size\;=\;4/3\pi r^{3}\;=\;\left(4*22*r^{3}\right)/\left(3*7\right)nm^{3},\\ & Then\;r\;=\;{\left(21*Size/88\right)}^{1/3}nm\;hence,Particle\;Diameter\left(D\right)\\ & =2{\left[\left(21*193.197/88\right)\right]}^{1/3}=\;2\left[3.59\right]\;=\;7.17nm\;where\;r:\\ & sphere\;radius,D:particle\;diameter\;=\;2r \end{array}$$

**Table 3.** Summary of TEM image analysis by ImageJ software.

Sample	Count	Total Area	Average Size	% Area	Mean
Z1	121	23376.879	193.197	48.383	245.457

### 3.3. Scanning electron microscopy (SEM)

The surface of the nanomaterials was observed by SEM (JEOL JSM-6510 LV) after coating the nanoparticles with a gold thin film before the SEM processing. SEM images of the selected nanocomposite samples as well as the parent blend were used to calculate the surface roughness parameters of the samples, which are summarized in Table 4. Three-dimensional images can be used to estimate the roughness parameters including average roughness (R_a_), root mean square roughness (R_q_), the maximum height of the roughness (R_t_), maximum roughness valley depth (R_v_), maximum roughness peak height (R_p_), the average maximum height of the roughness (R_tm_). Such measured parameters specify and support the suitability of the studied sample for specific applications

**Table 4.** Surface roughness parameters of **NC1**, **NC2**, **NC4**, **NC6** and its parent polymers.

Sample	CuO (ml)	Ra (nm)	Rq (nm)	Rt (nm)	Rv (nm)	Rp (nm)	Rtm (nm)
PVP	0	3.05629	3.81637	22.4715	11.2342	11.2373	16.2099
CMC	0	3.50983	4.82494	48.5574	19.7319	28.8254	23.6655
NC1	0	3.03238	3.78981	25.1440	9.93806	15.2060	18.2789
NC2	2	3.17854	4.13874	30.4637	14.9339	15.5297	19.9968
NC4	8	8.34343	14.8350	188.104	70.0965	118.008	56.4624
NC6	12	10.5764	19.8691	290.094	187.413	102.681	86.0970

**Figure 3.**
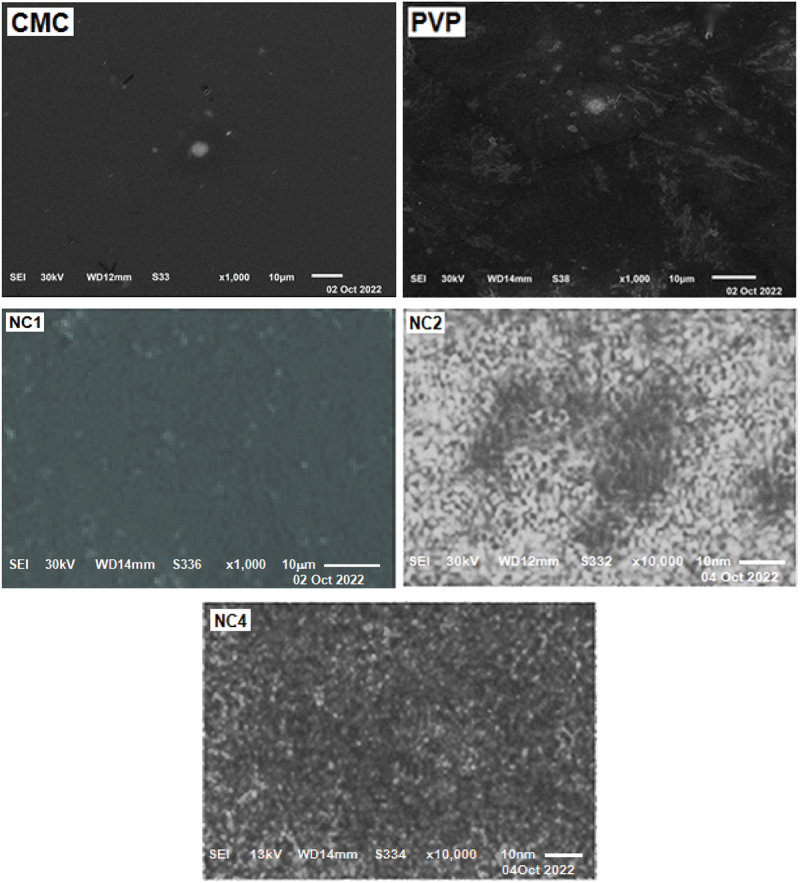
SEM images of selected nanocomposites CMC, PVP, NC1, NC2 and NC4.

Three samples of the nanocomposites of different concentrations of CuO NPs, namely NC1, NC2 and NC4, were selected to be micro-graphed as shown in Figure 3. SEM micrographs of the selected samples of different content of CuO showed that CuO NPs were more or less higher in roughness for NC4 than that for NC2 and NC1, which is expected based on the content of CuO NPs in the samples.

### 3.4. FT-IR spectroscopy

**Figure 4.**
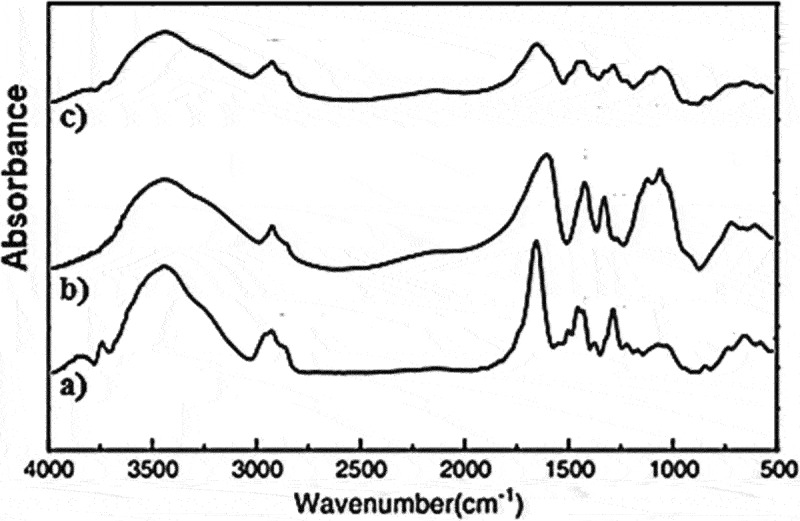
FTIR Spectra of **(a) PVP, (b) CMC**, and **(c)** CMC/PVP blend **(NC1)**.

Figure (4) shows the FTIR spectra of the PVP, CMC, and CMC/PVP blend. Figure (4a) shows PVP characteristic absorption bands of C=O stretching vibration at 1660 cm^−1^ [31–33]. Stretching and scissoring vibrations of the CH_2_-CH_2_ group at 2956 and 1448 cm^−1^ and a broad absorption band of OH stretching vibrations at 3446 cm^−1^ are recognized [34,35]. Figure (4b) shows CMC characteristic bands for OH stretching and a small band for C-H stretching at 3430 cm^−1^ and 2909 cm^−1^, respectively, while the strong band at 1605 cm^−1^ is related to the COO- group. Bands at 1420 and 1320 cm^−1^ are related to CH_2_ scissoring and OH bending vibrations, respectively. The band at 1060 cm^−1^ is assigned to CH – O – CH_2_ stretching [36,37]. FTIR spectra of the CMC/PVP blend **(NC1)** shown in Figure (4c) contains the characteristic bands of CMC and PVP, in which C=O of PVP appears at 1662 cm^−1^ suffering slight shift relative to the individual components, and the bands for COO- and CH_2_-O-CH_2_ groups of CMC appear at 1602 and 1062 cm^−1^ with relatively more shift than the individual components appeared at 1605 cm^−1^. The presence of such bands in **NC1** reveals the hydrogen bonding between CMC and PVP. Such interaction is based on reducing the absorbance of the OH group and increasing the broadness of the C=O group. The absorbance for COO- is significantly reduced and overlapped with the C=O group and exists as a shoulder. The absorbance for OH and CH_2_–O–CH_2_ groups is also reduced [38].

**Figure 5.**
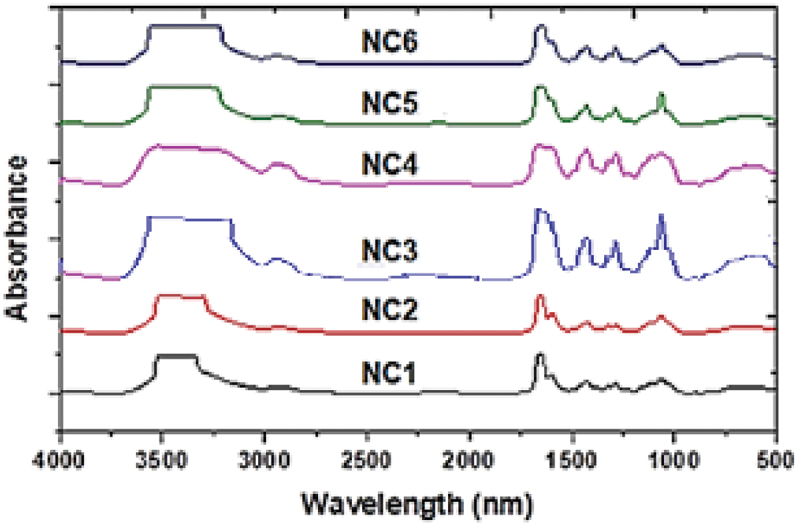
FTIR spectra of CMC/PVP blend **(NC1)** and its CuO NPs nanocomposites **(NC2-NC6)**.

FTIR absorption spectra of **NC2-NC6** samples of different CuO NPs content presented in Figure 5 shows similar behavior with an increasing trend with CuO NPs content. The appearance of a broad band centered at 3430 cm^−1^ corresponds to the O-H and N-H groups, while the absorbance hump at about 2930 cm^−1^ which increases with the concentration of CuO NPs within the matrix indicates a possible H-bonding between CuO NPs and the organic moieties in the blend. Sharp bands of the C=O group at 1725 cm^−1^ beside sharp intense bands at 1485 and 1020 cm^−1^ can be correlated with C – N and =C – H stretching vibrations, respectively. In addition, the presence of C – O, and C – C group vibrations in the region of 600–500 cm^−1^ and the absorption band at around 1725 cm^−1^ with a slight shift in the presence of CuO NPs as well as the increasing broadness of C=O and OH bands by addition of CuO NPs reflecting some interaction between CuO NPs and C=O groups in the matrix [39].

### 3.5. UV/Vis spectroscopy

**Figure 6.**
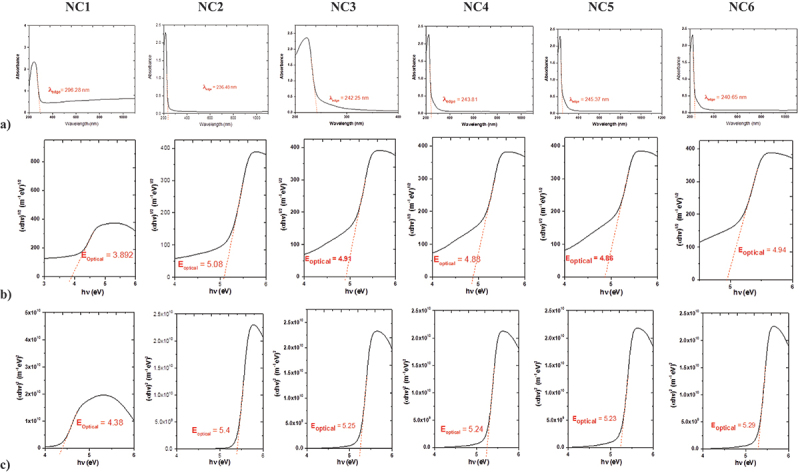
UV/Vis absorption spectra and Tauc Plot of CMC/PVP blend **(NC1)** and CMC/PVP/CuO NPs nanocomposites **(NC2-NC6)**.

The spectra associated with molecules that absorb energy within UV and/or visible regions to excite n-electrons from bonding or non-bonding states to higher anti-bonding orbitals can be used to estimate information about the electronic transitions of the studied material in different states. Other physical parameters can also be drawn from such spectral data, including the difference between higher and lower occupied molecular states (HUMO-LOMO) and the optical energy gap [40–42]. The absorption spectra of the blend sample shown in Figure (6-NC1) showed UV/Vis absorption maxima at 296 nm are related to the moieties in the polymer. Such absorption maxima is reduced to the range of 236–246 nm when CuO NPs are included within the blend matrix as shown in Figure (6) and reflect an interaction between CuO NPs and the blend matrix. The optical bandgap, **E**_**g**_, is determined from the absorbance spectra using Eq. 3.

(3) $$\left(\alpha h\nu \right)n\;=\;B\left(E\;-\;Eg\right)$$

where **B** is a constant related to the effective masses of charge carriers associated with valence and conduction bands, **E**_**g**_ the bandgap energy, E = hν the photon energy, and *n* = 2 or 1/2 for direct and indirect transition, respectively. The intersection of the slope of (αhν)^2^ *vs*. hν curve on the x-axis provides the bandgap energy of the sample. The *Tauc* plots of the system are displayed in Figure 6b. E value at α = 0for CMC/PVP blend showed intercepts at 3.89, and 4.38 eV for indirect and direct optical bandgaps, respectively. These values increased to the range of 4.85–5.08 eV and 5.23–5.40 eV for indirect and direct optical bandgaps, respectively, for **NC2-NC6** nanocomposites. These findings are summarized in Table 5 and correlated with the conclusions derived from the UV/Vis spectra.

**Table 5.** The Wavelength **(λ**_**edge**_) and indirect optical energy gap **(E**_**opt**_**1/2**) and direct optical energy gap **(E**_**opt**_**2**) for CMC/PVP blend and CMC/PVP/CuO NPs nanocomposites.

Sample	NC1	NC2	NC3	NC4	NC5	NC6
λ_edge_	296.28	236.48	242.25	243.80	245.37	240.65
E_Indirect_ (E_opt_^1/2^)	3.89	5.08	4.91	4.88	4.85	4.94
E_Direct_ (E_opt_^2^)	4.38	5.40	5.25	5.24	5.23	5.29

**Figure 7.**
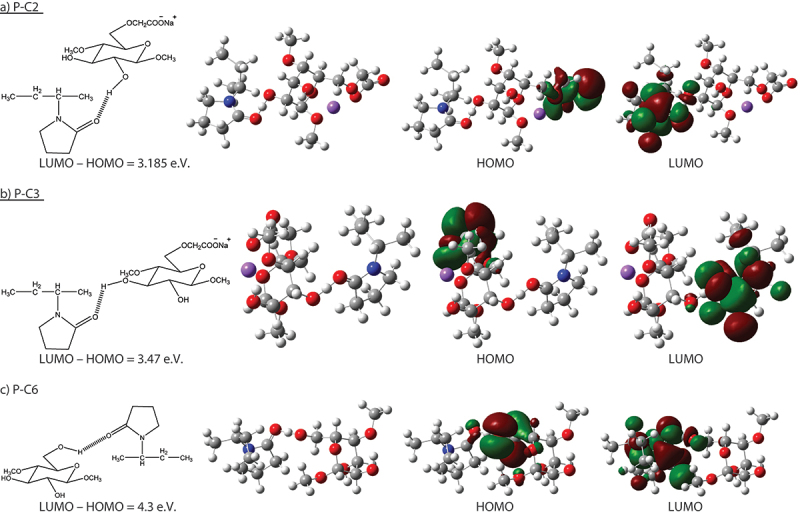
HUMO-LUMO calculations of the optimized 2D and 3D structural units in NC1 after H-bonding.

In addition, the optical energy gap can be calculated using the wavelength λ_edge_ in the intersection of the fundamental absorption edge, with the x-axis using the formula; E_g_ = *h* c/λ_edge_. Such information can be approved using the data obtained from optimized 2D and 3D structural units in combination with HUMO-LUMO data shown in Figure (7). Values of the optical energy gap (E_gap_) were calculated from the UV/Vis spectral data using the Mott and Davis formula [43,44] describing the photon energy (*h*υ) in terms of absorption coefficient (α). The HUMO and LUMO energy values were also calculated for the optimized structures and compared with those obtained from energy gap calculations.

The interaction between CMC and PVP in NC1 was investigated theoretically by Gaussian 09W 7.0 Software that is used for molecular calculations and to predict the most probable mode of interaction between two molecules or more. In the next context, there are different probable ways of interaction between the blend components through hydrogen bonding in different conformations. Comparing E_gap_ calculated from UV spectroscopic analysis of the prepared blend with those predicted by the Gaussian Software, one can predict the most conformation obtained from hydrogen bonding. A suggested interaction was mentioned early for only one mode of interaction [45–47]. Currently, many possible interaction modes can be suggested, but only a few can be of predominant probability supported by the Gaussian Software calculations.

### 3.6. Biological activity

Copper oxide nanoparticles play a key role in preventing fungal, bacterial, and microbial attacks on yeast and molds. Copper oxide nanoparticles effectively participate against the growth of bacteria, such as Bacillus subtilis, *Staphylococcus aureus*, and *Escherichia coli* by using the diffusion method. *E.coli* and *B. subtilis* show susceptibility against copper and silver nanoparticles. Copper oxide nanoparticles also participate in this type of study by using MRI. In the current study, nanocomposites were tested against the previously mentioned microorganisms using amoxycillin as a reference standard; hence, the results represent the relative activity of the investigated materials. The results are summarized in Table 6.

**Table 6.** Inhibition zone diameter of the investigated nanocomposites against *E. Coli* and *S. aureus.*

*Sample*	*E. coli* (mg/ml)		*S. aureus* (mg/ml)	
	Inhibition Zone Diameter (mm)	% Activity index	Inhibition Zone Diameter (mm)	% Activity index
NC1	NA	-	NA	-
NC2	7	26.9	6	25.0
NC3	4	15.4	10	41.7
NC4	11	42.3	12	50.0
NC5	14	53.8	14	58.3
NC6	12	46.2	16	66.7
CuO NPs	10	38.5	17	70.8
Amoxycillin	26	100	24	100

## 4. Conclusion

New nanocomposites of carboxymethyl cellulose, polyvinylpyrrolidone, and copper oxide nanoparticles (CMC/PVP/CuO NPs) with good properties were prepared, and their characteristics were explored to help predict suitable fields of applications. XRD indicated a change in crystallinity with an increase in the amorphous nature of the nanocomposites correlated with the doped amount of CuO-NPs and reflected on their physical characteristics. TEM imaging showed almost spherical shape domains with a slight deviation in shape and particle diameter that has been determined as 7.17 nm based on the averaging of the TEM image by using ImageJ Software developed at the National Institutes of Health (NIH). SEM imaging proved that the inclusion of CuO NPs induced surface roughness of the nanocomposites. The FTIR absorption spectra reflected interaction between CuO NPs and the polymeric matrix through interaction with C=O and other groups. The calculated optical bandgaps for the nanocomposites are in correlation with the conclusions derived from the UV/Vis spectra and supported by a theoretical prediction by using Gaussian 09W 7.0 Software. Finally, CuO NPs were proven to play an important role in preventing fungal, bacterial, and microbial attacks on yeast and molds, hence, CuO NPs effectively contribute against the growth of bacteria using amoxycillin as reference material.
